# Why are fewer women rising to the top? A life history gender analysis of Cambodia’s health workforce

**DOI:** 10.1186/s12913-019-4424-3

**Published:** 2019-08-23

**Authors:** Sreytouch Vong, Bandeth Ros, Rosemary Morgan, Sally Theobald

**Affiliations:** 1 ReBUILD/RinGs Consortia, Cambodia, Phnom Penh, Cambodia; 2 Department of International Health, Johns Hopkins Bloomberg School of Public Health, Baltimore, USA; 3 Department of International Public Health, Liverpool School of Tropical Medicine, Liverpool, UK

**Keywords:** Gender equity, Health workforce, Leadership, Life history, Cambodia

## Abstract

**Background:**

An adequate and qualified health workforce is critical for achieving Universal Health Coverage (UHC) and responding to the Sustainable Development Goals (SDGs). Frontline health workers who are mainly women, play important roles in responses to crisis. Despite women making up the vast majority of the health workforce, men occupy the majority of leadership positions. This study aims to understand the career progression of female health workers by exploring how gender norms influence women’s upward career trajectories.

**Methods:**

A qualitative methodology deployed a life history approach was used to explore the perspectives and experiences of health workers in Battambang province, Cambodia. Twenty male and female health managers were purposively selected based five criteria: age 40 and above, starting their career during 1980s or 1990s, clinical skills, management roles and evidence of career progression. Themes and sub-themes were developed based on available data and informed by Tlaiss’s (2013) social theory framework in order to understand how gender norms, roles and relations shape the career of women in the health industry.

**Results:**

The findings from life histories show that gender norms shape men’s and women’s career progression at different levels of society. At the macro level, social, cultural, political, and gender norms are favorably changing by allowing more women to enter medical education; however, leadership is bias towards men. At the meso organziational level, empowerment of women in the health sector has increased with the support of gender working groups and women’s associations. At the micro individual level, female facility managers identified capacity and qualifications as important factors in helping women to obtain leadership positions.

**Conclusion:**

While Cambodia has made progress, it still has far to go to achieve equality in leadership. Promoting gender equity in leadership within the health workforce requires a long vision and commitment along with collaboration among different stakeholders and across social structures. If more women are not able to obtain leadership roles, the goals of having an equitable health system, promoting UHC, and responding to the SDGs milestones by leaving no one behind will remain unattainable objectives.

## Background

An adequate and qualified health workforce is critical for achieving Universal Health Coverage (UHC) and responding to the Sustainable Development Goals (SDGs). Globally, there are more than 59 million health workers [1, 2]. Women make up about 70–75% of the global health workforce and deliver care for 5 billion people [3]. Despite this, gender inequities continue to persist which in turn hamper progress towards UHC [1, 4, 5]. Despite women making up the vast majority of the health workforce, the majority of women are situated in lower cadres of the health workforce and men occupy the majority of leadership positions [5–7]. The recent #LancetWomen initiative, for example, shows that women are underrepresented in senior management and governing bodies of global health organizations and policy making remains a male dominant process [4]. Inequities within the health workforce can imped entry into health occupations and overall career progression, with negative repercussions for health workers’ education, recruitment, retention and progression. Other negative effects include mal-distribution of workers, lower motivation and productivity, and experience of absenteeism [8], which in turn affect quality of health outcomes for patients [5].

Career advancement is particularly challenging in post-conflict states, where strategies and incentives to motivate workers, especially women, to stay and work in the health sector during and after crisis have been neglected. During crises, health workers often face constraints related to abduction, ambush, injury, displacement, and/or lack of a support system, all of which hamper career advancement [9]. Crisis periods also deplete the health workforce, due to health worker migration or death, causing a health worker shortage during the post-conflict period. In many crises, health workers are the frontline workforce implementing the emergency health system response. However, female health workers are particularly vulnerable during times of conflict and insecurity. The lessons learnt from Ebola in Sierra Leone show that the frontline health workforce played an important role in controlling the spread of the Ebola outbreak through dissemination of accurate information, undertaking surveillance, contact tracing, and promoting hygienic practices [10].

Cambodia experienced nearly three decades of civil war and conflict from 1970 to 1998. The Khmer Rouge regime that lasted from 1975 to 1979 led to the loss of up to 3.3 million people [11], and health workers were among the people executed for their professional skills. After the Khmer Rouge, there were only an estimated 40 physicians remaining in the country [12]. Full peace was not achieved in Cambodia until 1998. Rebuilding the health sector in Cambodia during the post Khmer Rouge period heavily relied upon women; this lasted until the 1990’s when health sector reform began. The reform includes the formulation of the first Health Workforce Development Plan (HWDP1:1996–2005), which aimed to address adequate production and equitable distribution of the health workforce to respond to the new health coverage plan [10]. The HWDP2 (2006–2015) aims to address the competency and management of the health workforce, followed by the third HWDP3 (2016–2020), which focuses on structure, size and composition of the workforce, including recruitment, employment, deployment, productivity and staff remuneration [13].

Recruitment of health workers into the Cambodia health sector has been improved after almost twenty years of health system strengthening and human resource development. By 2016, Cambodia health sector employs a total of 25,382 health workers (personal communication, humanresource department 2017). However, empowering women to enter leadership roles in the sector was still slow. Women are less likely to work at management positions or policy-making roles in all levels of the health system which includes the central, provincial, and operational health district and health center levels. As such, women are less likely to be in senior professional, managerial and policy-making roles and have less opportunities to be prepared for new positions [14, 15]. As a result, women took only less than 15% of leadership roles of the Ministry of Health (MoH), indicating the under-representation of women in the management structure of the health sector (Table 1).

**Table 1:** data of health workforce in health sector by gender (2010–2015)

	2010	2011	2012	2013	2014	2015
*Number health personnel*						
Total	18,113	18,302	18,814	19,721	20,668	20,954
Female	8072	8299	8698	9401	10,132	10,576
% of female	45%	45%	46%	48%	49%	50%
*Number of women in leadership position*						
Total	–	1097	1120	1209	1190	1214
Female	–	139	156	169	165	178
% of female		12.7%	13.9%	14%	13.9%	14.7%

Source: Human resource department, MOH 2017

Cambodia’s health system reflects the same challenge of health workforce gender segregation globally. Report by the World Health Organization (WHO) states, the health sector is “delivered by women and led by men” [3]. This situation raises several concerns that need addressing. Firstly, few women in managerial, policy and decision-making roles means few opportunities for women’s voices in health policies, strategies and management systems. Secondly, career progression for women to advance through the health sector that does not take into account women’s needs is clearly challenging with implications for gender equity. Within Lebanon, for example, normal life experiences of female health workers, such as pregnancy and childcare, become problematized due to their incompatibility with male work models that do not take life course events into account [8, 16]. Thirdly, given the social and cultural context, women prefer to be cared for by female health care workers. However, this care can often be met at the primary health care level, yet not at secondary and tertiary levels as there are few female doctors and specialists [17]. Better understanding and addressing these concerns are important for a responsive and equitable workforce.

Gender equity in the health workforce means both men and women are able to enjoy equal opportunities in, but not limited to, skill development and career advancement [8]. So far, very few studies on the health workforce have been conducted to explain the current gender disparities between male and female health workers in Cambodia. In addition, few studies have adopted a historical lens to understand the historical context and its impacts on the current gender inequity in health leadership in a post-conflict setting [18, 19]. Since women are dominant within the lower cadres of the current health system in Cambodia, but a limited number of women take up leadership roles, understanding women’s experiences and perceptions of, as well as barriers to, career entry and advancement in the past is important. Using a qualitative life history method, this paper adapts the framework of Tlaiss [16] to explore gender norms at the macro, meso and micro level to explain the current female leadership disparity in Cambodia health sector. The findings of this study may have wide applicability to other countries affected by conflict in where gender inequity issues in leadership have not been fully addressed due to gender norms, roles and relations and their historical legacies grounded in conflict.

## Methods

A qualitative methodology was used to explore experiences of health workers from their own perspectives. In-depth interviews with health managers deployed a life history approach, using an interview guide (Additional file 1) developed to capture major life events and career history of health managers for this study. This enabled researchers to explore the gendered experiences, career trajectories, motivation and barriers to the decisions of male and female managers to enter, progress, and advance their clinical skills and career through time, and its implication for women’s contemporary experiences of leadership. The life history method allows researchers to elucidate a person’s micro-historical (individual) experiences within macro-historical perspectives through time [20]. In this case - pre, during, and post conflict. The use of a life history approach was important in two ways; firstly, it allowed researchers to capture and analyse the dynamics of gendered decision-making of participants in different political periods, and secondly, it helped empower participants as they were able to narrate using their own voices [20].

### Sampling approach

Fieldwork was conducted in February 2016 in two operational heath districts (OD) (Battambang and Moung Russei), in Battambang Province, Cambodia. The selection of the two ODs was based on the high proportion of female managers at health centers and district offices. Using a ‘positive deviance’ approach, a total of 20 participants (14 females and 6 males) who demonstrated a leadership progression were purposively recruited from the two operational districts. A positive deviance approach enabled us to document best practices, effective strategies or robust innovation of successful female leaders with the aim of promoting widespread uptake of such practices and to address the gender gap in health sector leadership [21]. Participants were purposively selected based on a combination of five criteria: age, service date, clinical skills, position, and leadership progression. Selected participants were age 40 or above and started their career during the 1980s or 1990s so that they were able to reflect on their experiences through time. However, we also happened to select one young female health center manager who was 30 years old, having taken up a leadership position in the late 2000s.

### Analysis and conceptual framework

**Fig. 1:**
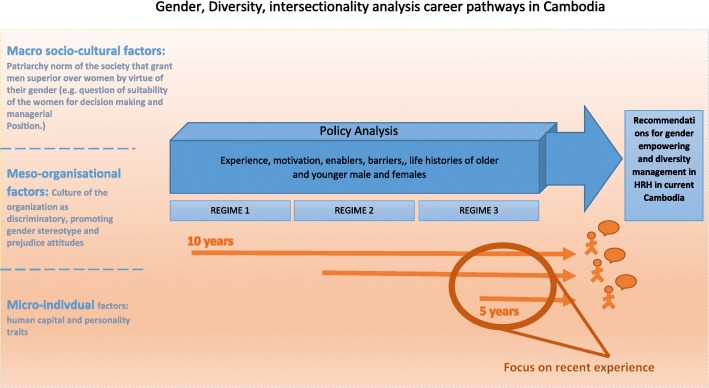
Gender analysis on health workers’ career pathways in Cambodia

Interviews were recorded and transcribed in Khmer (official Cambodian language) and then translated into English. The research team employed inductive thematic analysis. Each transcript was analysed looking for different perspectives between female and male managers and information related to the effects of gender norms, roles, and relations on motivation and barriers at individual, household, community, or institutional levels. Themes and sub-themes were developed based on the available data and informed by Tlaiss’s (2013) social theory framework in order to understand how gender shapes the career of women in the health industry. Figure 1 shows the framework of analysis. This study received ethical approval from the National Ethics Committee for Health Research in Cambodia No 275 NECHR.

## Results

Health workers were asked about their experience to enter, progress and advance their clinical skills and career progression in the health sector from the 1980s to 2016. Table 2 describes the career pathway of the health workforce emerging from the life history analysis of our 20 participants. Using the framework of Tlaiss (2013), three themes emerged from the analysis: (1) while the macro social, cultural, political and gender norms are shifting, gender equity at home is still needed; (2) while meso organizational health sector support for women has increased, it is still insufficient; and (3) at micro individual level, women’s capacity and confidence in the workplace has improved through time.

**Table 2:** Career pathway of health workforce in Cambodia (1980s to 2016)

	1980s	1990s	2000–2016
Context	Post Khmer Rouge regime, K5 (the period between 1985 and 1989 when the government set a plan to seal Khmer Rouge guerrilla infiltration routes into the central Cambodia) (start rebuilding health sector)	Paris Peace Accord; first election held in 1993; health sector reform	Full peace achieved in 1997; continuation of health sector reform (user fees, Health Equity Fund, health coverage plan, health workforce development plan)
Entering medical school	▪ Government’s demand for HWs to respond to needs of health service after KR ▪ Recruitment: based on the rapid response to the needs of health care services	▪ Government’s policy encouraged people to enter health workforce ▪ Recruitment: based on the need of health care services and personal interests in medical field	▪ Strong interest from individuals for medical education (wider awareness of medical education) ▪ Presence of private medical college ▪ Recruitment: based on needs of health services and enhancing quality of health workforce
Serving health workforce and leadership	▪ Women were discouraged to enter workforce: insecurity and gender norms, no restrictions for men ▪ Social recognition & appreciation of female health workers in staff-shortage/remote/under conflict areas	▪ Stigmatization of female workers on night shift, working far away from home ▪ Less support from male colleagues	▪ No social stigmatization on girls entering medical education ▪ Asymmetrical gender norms: expected roles of women to undertake household chores and child rearing ▪ Institutional support: presence of Gender Working Group in sub-national level
Advancing clinical skills	▪ Existence of policy to support the continuation of medical education but only: • Single women • Married women but not having children yet • Married with support from husband	▪ No clinical advancement among managers in this period ▪ Lack of institutional support for clinical progress ▪ Women are obligated to undertake family and child rearing responsibilities	▪ Married women were able to continue their medical education ▪ Presence of male involvement in sharing domestic chores and child raring

### While the macro social, cultural, political and gender norms are favorably changing, gender equity at home is still needed

Most life histories show that between the 1980s–1990s women faced social, cultural and political constraints and restrictions in entering and studying in medical schools or performing their medical duty. However, since the 2000s social and cultural stigmatization against women to enter and work within the medical profession reduced.

In the 1980s, women were often discouraged from entering medical school because of security issues and beliefs around women’s domain being around the home, women’s lack of intelligence, or women’s role as wives taking precedence over their studies. Poverty placed more burden on females in the family (particularly older daughters), and opportunities to receive even general education were given to men (sons) in this period:

*“Even after I passed the exam, the elders in my village had gathered and talked. They discouraged me. They said I have to have a good memory to study medical subject, so on and so forth”* (F_46)

Men were found to have no restrictions in terms of going out or studying far from home. As one male participant recalled:

*Q: “Do you think it was easier for men to travel outside [the country] at that time? A: Of course. Men could go anywhere… [my parents] didn’t worry about me. I could go anywhere because I was a man”* (M_64)

Since the 2000s, gender norms that discouraged girls to study away from home due to security problems, or study medical subjects because of perceived lower intelligence is not relevant anymore. However, the expected roles of women in the household, such as childcare and household chores still existed. Like the previous periods, women had to juggle and find assistance to manage their situation. Finding support from partners and family members or hiring labour to assist them helped reduce burdens on women:

*“At that time, I had a helper who helped with household work and care taking… I often said to her that please help to look after your younger brothers and sisters [my children]”* (F_59)

Even so, there is still a challenge to achieving gender equity within the home. Female managers (also health providers) found it difficult to combine domestic chores, including breastfeeding and taking care the elderly in the family, with their managerial roles. In some cases, the female managers needed to leave their job early, bring children to work or take a break from their job for a period.

*“I took 2 years suspended from work as my dad was in severe sickness and no one took care him… [taking care of parents] is the task of daughter, especially when we are not married. Daughters love their parents more [than son], the son is busy with their own family.”* (F_55)

Male managers corroborated this view in that they believed women were responsible for household chores. They emphasized that roles between men and women within the family were defined and that women are supposed to work inside household, while men are in charge of business outside the household:

*“It’s the roles of women to take care home, it must be like that! In fact, we need to divide the tasks, household chores and the work outside…, if we mix it up, we can’t do many things!”* (M_51)

Female facility managers emphasized the need for support from relatives and spouses. Some female managers appreciated the support from their spouse to share child rearing and domestic chores, allowing more time for female managers to concentrate on work:

*“My husband is often the one who cooks the rice…Once we finish eating, I do dishes and return to work… it does go against the gender norms, but my husband understands my condition.”* (F_58)

Societal views at the macro level after the 2000s were still mainly biased towards men and accustomed to the norms that women are not suitable for decision-making roles.

*“It’s a cultural barrier. Some men still think women are not strong yet and still weaker than men.”* (F_46)

In addition, men are often considered as having better strategic vision and being more suitable in leadership roles. Female managers also felt that their voice was less respected, that their age influenced the trust and respect they received, and that they were not perceived to the same degree of competency that men had:

*“[In the meeting] mostly they accept men’s ideas because they believe that men have a strategic vision.”* (F_44)

“*Some people disappointed [with me] as he is older [than me] and I become his chief”* (F_50)

To cope with this, a few perceived that female leaders need to work extra hard to improve their outputs, so that respect and trust could be gained.

### While meso organizational health sector support for women has increased, it is still insufficient

In the past, there was no gender-sensitive policy at the organizational level that supported women to work and serve in the health sector. After the Khmer Rouge, government health policy focused on staff recruitment in response to needs, however, there was no focus on gender equity, and no efforts to improve the social recognition of women’s roles in health sector.

During the K5 period in the mid-1980s, while the number of female health workers was already small, women had to be on duty within conflict-affected areas, including stationed within forests with groups of militaries. This has placed women in a challenging position as societal gender norms were that women should not stay away from home or community. Female health workers had to confront the stigmatized perceptions of their families or villagers and encountered security problems. Only peer support motivated them to stay in the job at that time.

Both male and female health workers did not receive many chances to advance their clinical skills in the 1980s and 1990s. The lack of encouragement by managers was related to the staff shortage at the workplace. For example, it was difficult for managers, especially during the 1990s, to send staff to study as they would lack the staff required to perform tasks within the facilities:

*“I also wanted, but the health center lacked staff to work. If I went to study, there would be insufficient staff providing services at health center. Health center just consisted more staff in 1998-1999. Before that, there were only 3 staff.”* (F_44)

In contrast, from the 2000s onward, there was support for gender mainstreaming. At the meso organizational level, participants reported the presence of a gender-working group and the establishment of a women’s association within the workplace to support female health workers. Women also mentioned having gender training available to build capacity and confidence of women to showcase to their managers. During this time government policies and quotas promoted women’s entry into leadership positions.

Some superiors (mainly male) also addressed problems and paved the way for women to progress in their career. All female facility managers acknowledged strong support from the (mostly male) head of the local institution in providing advice, particularly in the early stage of their leadership journey.

*“…I never expected a man, being a leader, valued women like this because generally man is likely to get promoted. However, my chief was different from other; he promoted women.”* (F_44)

### At the micro individual level, women’s capacity and confidence in the workplace has improved through time

Most female participants claimed their capacity, confidence and self-motivation had improved in the current period. In the past, they mentioned self-interest, hard work and determination were the key enablers to support them to enter and work in health sector, but in the present, female facility managers identified capacity and qualifications as important factors in helping women to obtain leadership positions. Women needed to pursue their degree and advance their skills to a higher level to be able to gain trust from their male co-workers. Another female manager emphasized “willing to try and take risk” as a way to motivate themselves to take up a leadership position.

*“Men won't listen to women because those women do not yet have enough capacity. If they have enough capacity, they won’t feel so.”* (M_64)

*“For leadership, if the women are smart and having high knowledge they can lead! But if some women are not smart, no capacity, how can she be a leader?”* (F_55)

**Fig. 2:**
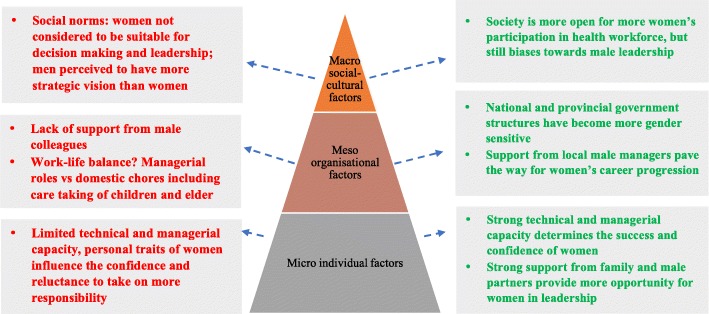
Barriers and enablers to women’s leadership in heath workforce

As the above analysis demonstrates, female leaders have faced both barriers and enablers throughout their career pathway to enter, progress, and advance their careers. Figure 2 summarizes the key barriers and enablers to women’s leadership based on the 20 participants in the health workforce and how these play out at the macro, meso, and micro levels.

## Discussion

This paper explores the career pathway of health workers in Cambodia assessing how past and present factors underpin the current gender disparity in leadership within the health sector. The life history analysis shows how the current disparity of female leadership in the heath sector is determined by social, cultural, political, and gender norms, in addition to organizational and household norms; and that these factors interplay and have changed through time to affect women’s career progression.

Our study found that the current shortage of women in leadership today is partly influenced by the macro political, social, cultural, and gender norms from the past. Both men and women were constrained during the crisis period to join the profession and perform their roles due to high levels of poverty, limited jobs and military conscription. Though unsafe working conditions affected health workers in general [5], gender norms, linked to and exacerbated by conflict, meant that women faced additional barriers to entering medical school and advance their skills. At work, women played active roles in helping to save the lives of people in conflict-affected areas; however, they were more vulnerable than men in terms of personal safety, privacy and security. Women encountered community stigma if they spent periods away from home. Similar findings have been found in Sierra Leone, particularly in relation to barriers which demotivated female staff to work in rural areas [22].

At the current macro level, societal and gender norms are more open to accepting women within the public workforce; yet leadership is still biased toward men. Women still face discrimination within leadership positions. This may due to gendered and social norms that constrain women’s participation in policy and decision-making positions. Having more women represented in leadership and policymaking positions is a crucial step for gender-transformative policy in all level of health system [4].

Household and reproductive labour work is still perceived as women’s work. Women’s dual roles within and outside the home constrain their ability to enter and progress within leadership positions. The recent study shows that women in the health workforce contribute to about USD 3 trillion annually to global health, half of which is in form of unpaid care [4], which is often unrecognized by society. Our study shows only “positive deviance” women, those who could commit, stay and work in the health sector, and despite discrimination they received from family and the community, managed to reach leadership positions and progress in their career. According to Witter et al. [18], these individuals often have a high level of intrinsic motivation. Individuals who are encouraged and motivated are often able to persevere and challenge barriers at the micro- and meso-level in order to reach their goal [16].

Balancing domestic chores and public work, including being a health provider and manager of a health facility, is a critical factor which enables current female leadership to enter and remain in their positions. A recent study confirms that balancing work and family, including addressing childcare needs, is a key factor underpinning under-representation of women in medical leadership [23]. We found that this barrier was less prevalent with male managers. Strong support from male partners at the meso organizational level provided more opportunities for women to enter leadership roles. As a result, gender equity within the home is necessary towards achieving gender equity within the workplace. A previous study highlighted the importance of achieving gender equity in the home as an integral part of achieving gender equity in society [24]. Our study demonstrates how approaches to support equitable gender relations both at home and the workplace are important to the professional development of female managers. Connecting action on gender-equity to health workforce and decent work agendas, as well as within the home, is a win-win situation that could contribute to gender transformative agenda [4].

Gender mainstreaming within Cambodia’s health workforce has improved through time. However, women’s career progression and entry into leadership positions is still constrained by uneven organizational support. As a result, women are struggling to engage and utilise their full capacity in the health sector largely due to the lack of gender-sensitive policies [25]. The findings showed that supporting factors for career progression toward leadership from male managers, especially in the very early stage of career and leadership at the meso organizational level, is important. Similarly, a study of engaging men to support women in science, medicine and global health highlights that the collective privilege of men and their power over women in society is an underlying factor contributing to less female representation in science, including medicine and global health [26]. The same study also suggested that male involvement to support women in career progression requires a systemic approach, particularly at the institution level [26].

Finally, having organizational support at the meso level is not enough. Organizational cultures (embodied by male and female managers and role models) that value women’s capacity and qualifications and believe in the agency of women leaders are key towards achieving gender equity within health system leadership. To build such organizational cultures, [27] suggest to have programs that encourage everyone within the organization to feel personally responsible for change. This should also start with group’s leaders (especially male heads within the organization) committing to the change and encouraging others to follow [27]. Having high clinical and management skills at the micro individual level also makes women more confident to enter leadership roles. The influence of gender norms at the macro level which see women as more suited for administrative roles that support male counterparts and less suited for leadership roles are still the barrier for women in leadership.

Resilient health systems require a resilient health workforce. Without addressing gender inequity within the workforce and leadership, the health workforce will be unable to respond to and cope with shocks and crises. Recognizing the diversity of the workforce and acknowledging and addressing the constraints of gender norms in different layers of the health system helps create a more resilient health system [8] that’s able to respond to the different needs of men and women health workers and the communities they serve. Without addressing systemic gender bias, opportunities for advancement within health system and promoting women into decision-making positions will prove challenging [4]. Promoting gender equity in the health workforce requires gender mainstreaming processes in recruitment, retention and upward mobility of women in all cadres, including preservice and in-service training opportunity [4].

### Study limitations

More female than male managers were purposively selected for interviews, as we wanted to explore women’s perceptions, barriers and constraints to their leadership experiences. Only one young manager from the post-conflict period was included. We did not include women and men from all levels of the health system (for example those not in leadership positions). Rather using a positive deviance approach to sampling, we included both women and men who experienced successful progression, rather than those who had shown no progression, in management and leadership level in the post Khmer Rouge period. These leaders were recruited for the study because we aimed for in-depth understanding about the experiences of those who had progressed in leadership, learn from their experiences and hence contribute to the gap in the literature on health leadership research from a gender perspective in Low and Middle Income Countries.

## Conclusion

Female managers’ leadership progression was shaped by their own history, political context and social factors, including gender norms. Our study confirms that gender norms intersect with other social determinants to shape women’s career pathways and trajectories. Promoting equity in leadership within the health workforce requires systemic collaboration of different stakeholders. Ensuring more women are able to take on leadership positions cannot be achieved in the short term; it is a long-term process that requires support to mainstream gender at the micro individual level, meso organizations level and macro social and cultural level. At the meso institutional level and macro social level, including the community, social behavior changes to alter harmful gender norms, roles, and relations is required. Gender roles and norms within the family and community need to be challenged and changed. At the micro individual level, self-motivation, support from family or spouse, and appropriate capacity and qualifications of female providers all empower women to break through the glass ceiling to work in leadership roles.

Within conflict affected countries with similar entrenched gender norms, promoting gender equity in the health workforce requires a long vision and commitment, particularly having male support and involvement at different levels of society (including policy, institution, community and household level). Having female role models and mentorship programmes for junior health workers would also be crucial in many contexts. Without having more females in the workforce and leadership positions, the workforce system may not be resilient and responsive to the needs of the population, particularly women. If more women are not able to obtain leadership roles, the goals of having an equitable health system, promoting UHC, and responding to the SDGs milestones by leaving no one behind will remain unattainable.

## Data Availability

It is not possible to publicize the data from this study. The data that support the findings of this study could be available from RinGs but restrictions apply to the availability of these data, which were used under license for the current study, and so are not publicly available. However, the data could be available from authors upon reasonable request and with permission of RinGs.

## Abbreviation

HWDP: Health Workforce Development Plan
MOH: Ministry of Health
NECHR: National Ethics Committee for Health Research
OD: Operational Health District
UHC: Universal Health Coverage
